# The Performance of *Pleurotus eryngii* β-Glucans on Protein Digestion and the Release of Free Amino Acids in the Bloodstream of Obese Adults

**DOI:** 10.3390/foods14152649

**Published:** 2025-07-28

**Authors:** Charalampia Amerikanou, Stamatia-Angeliki Kleftaki, Aristea Gioxari, Dimitra Tagkouli, Alexandra Kasoura, Stamatia Simati, Chara Tzavara, Alexander Kokkinos, Nick Kalogeropoulos, Andriana C. Kaliora

**Affiliations:** 1 Department of Nutrition and Dietetics, School of Health Science and Education, Harokopio University, 17676 Athens, Greece; matina.kleftaki@gmail.com (S.-A.K.); dtagkoul@hua.gr (D.T.); alexkasoura@gmail.com (A.K.); htzavara@med.uoa.gr (C.T.); nickal@hua.gr (N.K.); akaliora@hua.gr (A.C.K.); 2 Department of Nutritional Science and Dietetics, School of Health Science, University of the Peloponnese, Antikalamos, 24100 Kalamata-Messinia, Greece; a.gioxari@uop.gr; 3 First Department of Propaedeutic Internal Medicine, Laiko General Hospital, National and Kapodistrian University of Athens School of Medicine, 11527 Athens, Greece; simatistemi@gmail.com (S.S.); akokkinos@med.uoa.gr (A.K.)

**Keywords:** *Pleurotus eryngii*, aromatic amino acids, gas chromatography–mass spectrometry, obesity, metabolic syndrome

## Abstract

*Pleurotus eryngii* is an edible mushroom with previously characterized β-glucans. Its potential to ameliorate postprandial glycemia and regulate appetite at the postprandial state has been previously shown. However, its effect on protein digestion remains unexplored. We aimed to investigate the effect of baked *P. eryngii* with a known β-glucan content (4.5 g) on plasma free amino acids of patients with central obesity and metabolic abnormalities at a postprandial state. In this acute, randomized controlled cross-over study, thirteen healthy male volunteers consumed one meal that was prepared with *P. eryngii* and one control meal; each meal was separated by one month. Blood was collected, and plasma was isolated at different timepoints before and after the consumption. Gas chromatography–mass spectrometry was used to quantify 24 free amino acids in the plasma samples. The area under the curve with respect to increase (AUCi) was computed, and the AUCi for aromatic amino acids was found to be higher after the consumption of the control meal compared to the *P. eryngii* meal (*p* = 0.027 for phenylalanine, *p* = 0.008 for tyrosine, and *p* = 0.003 for tryptophan). The above novel findings suggest that the β-glucans present in *P. eryngii* mushrooms are potential modulators of AA release into the bloodstream.

## 1. Introduction

Metabolic syndrome (MetS) is a cluster of metabolic abnormalities, including dyslipidemia, insulin resistance, hypertension, and central obesity [1]. The prevalence of obesity has risen dramatically in the past 50 years, and obesity is an important health challenge since it is a major risk factor for type 2 diabetes (T2D), hypertension, and cardiovascular disease development [2]. Recent advances support the importance of dietary strategies in the prevention and treatment of central obesity and MetS. Specific foods and food components as well as some nutraceuticals have been suggested as promising alternatives for the management of MetS and its components [1].

Edible mushrooms, such as *Pleurotus eryngii* (*P. eryngii*), have an important nutritional value and contain a number of bioactive compounds, including phenolic acids, resveratrol, triterpenic compounds, ergosterol, and β glucans [3]. In particular, according to current findings based on NMR metabolomics, *P. eryngii* has a mixture of alkali-soluble (1➔3 ) and (1➔ 6)-β-d-glucans, the alkali extract from *P. eryngii* comprising at least three structurally distinct polymers, hypothesized to be structurally related [4]. In previous studies from our group conducted in metabolically unhealthy humans, we showed that consumption of *P. eryngii* (containing 4.5 g of b-glucans) as part of a meal with a known protein and carbohydrate composition could ameliorate postprandial glycemia and appetite and could regulate ghrelin at the postprandial level [5]. Likewise, the daily consumption of *P. eryngii* for three months (equal to 3.5 g b-glucans daily) was shown to regulate glucose levels and anthropometric measurements as well as decrease LDL, SGOT, IL-6, and ox-LDL levels in people with obesity [6].

Amino acids (AAs) play an important role in the insulin signaling pathway, and their profiles are different among obese and insulin-resistant patients [7]. Hence, a number of studies have reported that blood AAs are significantly increased in T2D and may lead to the development of obesity [8,9]. More specifically, branched-chain AAs (BCAAs), including leucine, isoleucine, and valine, are increased in patients with obesity and are related to abnormal glucose and lipid metabolism [10,11]. Aromatic amino acids (AAAs), especially phenylalanine and tyrosine, are associated with metabolic disorders [12]. In a large cohort study, nine AAs, including phenylalanine, tyrosine, tryptophan, alanine, isoleucine, leucine, and others, were associated with a decrease in insulin secretion and an increase in fasting and 2 h glucose levels [13]. In the same study, tyrosine, alanine, isoleucine, aspartate, and glutamate were significantly associated with an increased risk of T2D incidence [13].

Regarding protein digestion and release of free AAs, it has been shown that the in vitro protein digestibility of a plant protein (either lentil or pea protein) is limited by approximately 30% in the presence of barley β-glucans. This is probably attributed to the protein–barley β-glucan complex formation having decreased transmittance and surface charge and increased particle size [14]. On the contrary, in other studies, the presence of oat glucans in yogurt led to faster proteolysis and a higher percentage of free amino acids in an in vitro-simulated digestion [15,16]. Apart from the potential impact on protein physicochemical properties, β-glucans have been shown to alter gut microbiota and AA metabolism in inflammation-mediated diseases [17].

While AA blood levels in obesity are linked to decreased insulin sensitivity and β-glucans may affect protein digestion and AA metabolism, data derived from food interventions aimed at controlling AA levels in people with obesity are absent. Thus, the main objective of this study is to evaluate the effects of *P. eryngii* mushrooms, rich in a mixture of (1➔ 3) and (1➔ 6)-β-d-glucans, on the plasma free AA profile of patients with metabolically unhealthy obesity at a postprandial state. As studies of postprandial responses of natural products rich in glucans usually focus solely on glycemic outcomes, our study intends to focus on other parameters associated with glucose regulation, such as free AAs.

## 2. Materials and Methods

### 2.1. Study Design

This study was performed in a subset of adults with central obesity and metabolic abnormalities who participated in an acute, randomized controlled cross-over study registered under the ID number NCT04444219 with the Clinicaltrials.gov platform [5]. The protocol was approved by the Institutional Review Board of Laiko General Hospital (ID protocol: 4931/22.03.2020). The Helsinki Declaration principles and the Data Protection Act 1998 were adhered to. All participants, after being informed about the objectives and procedures of the study, signed a written consent after agreeing to participate.

Eligible participants had to visit the premises of the “Diabetes Laboratory” of the First Department of Propaedeutic Internal Medicine, Medical School, National and Kapodistrian University of Athens (Greece) on two separate days for testing. This study comprises two days of testing after consuming one meal with *P. eryngii* and one control meal, respectively, separated by one month and in a randomized order. On each of the test days, participants were required to fast overnight. An intravenous catheter was placed into a peripheral vein, and blood samples were taken. Then, the participants consumed a breakfast consisting of 40 g commercial yellow cheese combined with white bread and 15 g of baked *P. eryngii* or the control breakfast consisting of the same quantity of cheese and bread, no mushrooms, and only 6–7 cherry tomatoes to achieve the same caloric content. A total of 15 g of baked mushrooms was selected to ensure an intake of approximately 4.5 g of glucans in the test meal, based on measurements from our previous study [5]. This dose was selected as most studies report health benefits in doses between 2 and 6 g, while doses exceeding this range have documented minor side effects. The time of the meal did not exceed 10 min. The nutritional composition of the two breakfasts is presented in Table 1. In detail, each meal was evaluated for its protein, fat, and available carbohydrate content, expressed in grams, as well as total energy, measured in kilocalories. Both meals were similar in nutritional profile. Protein content differed only slightly (23.0 g vs. 25.5 g), as did fat (12.7 g vs. 13.3 g) and available carbohydrates (55.4 g vs. 52.6 g). This minimal variation leads to nearly identical energy values—427.9 Kcal for the control and 432.1 Kcal for the *P. eryngii* meal. During the test, subjects could drink up to 500 mL of water if needed. More details on the preparation of the snack and on the study protocol and procedures have already been extensively described in the main publication of the study [5].

Subjects were advised not to drink alcohol or exercise the day before the trial and to keep stable dietary habits and physical activity levels throughout the study. Experienced dietitians provided consulting on how to preserve their lifestyle habits and were monitoring protocol adherence through validated questionnaires before each intervention. More specifically, dietary intake was evaluated with a 24 h recall record (4 non-consecutive days) and was analyzed with Nutritionist Pro™ v 7.9 (Axxya Systems, Stafford, TX, USA) software. Physical activity was assessed with the International Physical Activity Questionnaire Short Form, a 7-day recall instrument that estimates physical activity in MET-min/week. No statistical differences were observed on energy intake and physical activity score between the two interventions; therefore, all subjects were included in the analysis.

### 2.2. Participants

Subjects with central obesity, as determined by greater than 94 cm waist circumference (WC) in males and greater than 80 cm in females, were recruited. Non-eligible subjects were pregnant or lactating females as well as subjects with thyroid disease, alcohol abuse, or with known psychiatric or mental disorders. All participants were advised to abstain from alcohol and vigorous exercise on the day before each trial. They were also instructed to keep their dietary habits and physical activity levels consistent until the completion of the study.

### 2.3. Clinical Assessment and Anthropometric Measurements

Detailed medical history (personal and family) was obtained. Body weight was measured to the nearest kg with a flat scale in light clothing without shoes. Height was measured to the nearest cm using a stadiometer (Seca Mode 220, Hamburg, Germany). Body composition was analyzed with bioelectrical impedance analysis (Tanita BC-418, Tokyo, Japan) in order to calculate body fat, fat-free mass, and visceral fat level. Hip circumferences (HC) and WC were measured with a flexible, non-stretch tape on minimal clothing.

### 2.4. Biochemical Analyses

Standard blood withdrawal was performed (20 mL) at baseline and at 30 min, 60 min, 90 min, 120 min, 150 min, and 180 min after finishing each breakfast meal. Blood samples were centrifuged at 20 °C and 3000 rpm for 10 min for serum isolation. Biochemical markers were measured in serum with the use of a Cobas 8000 analyzer, an automatic biochemical analyzer (Roche Diagnostics GmbH, Mannheim, Germany): glucose; insulin; total cholesterol (TC); low-density lipoprotein (LDL); high-density lipoprotein (HDL); triglycerides (TG); alanine aminotransferase (ALT); aspartate aminotransferase (AST); glutamyltransferase (γ-GT); alkaline phospatase (ALP); lactate dehydrogenase (LDH); urea; uric acid; creatinine; iron (Fe); ferritin; albumin; and C-reactive protein (CRP). Homeostatic Model Assessment for Insulin Resistance (HOMA-IR) was calculated as follows: Fasting Glucose (mg/dL) × Fasting Insulin (μU/mL))/405. Plasma was used for the quantitative analysis of free amino acids using gas chromatography–mass spectrometry (GC/MS).

### 2.5. Free Amino Acids Quantification

#### 2.5.1. Derivatization of Free Amino Acids

Free AAs were derivatized, as described by Kaspar et al. (2008), with minor modifications [18]. In detail, 50 μL of plasma sample along with 20 μL of stabilization solution (aqueous solution of 10% iso-propanol, 0.1% phenol, and 2% thiodiglycol) were pipetted into glass test tubes. Then, 10 μL of norvaline solution 200 μΜ was added as an internal standard, followed by 120 μL of NaOH 0.33 M and 50 μL of picoline in propanol (20:80). Afterwards, the mixture was vortexed, and 50 μL of propyl chloroformate in chloroform (chloroform:propyl chloroformate:isooctane 60:20:20) was added. The mixture was emulsified by vortexing for 10 s and then left at room temperature for at least 1 min. The mixture was re-emulsified by vortexing again and then allowing the reaction to proceed for at least one additional minute. Finally, 250 μL of iso-octane was added. The mixture was vortexed for 10 s and then left to equilibrate for one minute or more until it was separated into two layers. Aliquots of 100 μL of the upper organic layer were transferred into GC vials, sealed, and analyzed immediately.

#### 2.5.2. GC/MS Analysis of Free Amino Acids

A gas chromatograph (Agilent GC 6890N, Waldbronn, Germany) coupled with a mass selective detector (HP5973, Electron Impact, 70 eV), split–splitless injector, and an auto sampler (HP7683) was used for the free amino acids analysis. Two µL of derivatized samples was injected into the GC at a split ratio of 1:15. An amino acids analysis dedicated column (length = 10 m, internal diameter = 0.25 mm, film thickness = 25 µm) was used, allowing for separation (Phenomenex Zebron ZB-A, Phenomenex^®^, Torrance, CA, USA). The carrier gas, high purity helium, was at a constant flow of 1.1 mL/min. The oven program along with the temperatures used were as described in the EZ:faast manual instructions (Phenomenex^®^, Torrance, CA, USA). The temperatures of the transfer line and of the injector were kept a 250 °C and 340 °C, respectively. The initial temperature of the oven was set at 110 °C and then increased to 320 °C at 30 °C/min for 3 min. A selective ion monitoring GC–MS method was applied for detecting and quantifying free amino acids, based on the ±0.05 RT presence of target and qualifier ions at the predetermined ratios, together with the electronic library “Agilent.L”, which was provided with the Ez:faast (Phenomenex^®^, Torrance, CA, USA) (Table S1). Quantification was performed using norvaline as the internal standard, and five points’ reference curves were constructed for each AA by multistandard AAs solutions provided by Sigma-Aldrich (Darmstadt, Germany).

### 2.6. Statistical Analysis

A sample size calculation estimated that, for an effect size of 0.8, an alpha level of 0.05, and an estimated power of 80%, the required sample size for a paired sample *t*-test was 13 subjects. For the purposes of this study, we used a subsample from the initial population [5] to measure the levels of free amino acids in plasma after applying an additional inclusion criterion—presence of a sufficient biological sample and non-hemolytic plasma. Statistical analysis was conducted using SPSS statistical software (version 26.0, SPSS, Inc., ΙΒΜ, Chicago, IL, USA). Continuous variables are presented as mean and standard deviation (SD) and qualitative variables as counts (%). For all amino acids, measured in different timepoints, the area under the curve with respect to increase (AUCi) was computed. AUCi values were compared between the two meals via the paired *t*-test. All *p*-values reported are two-tailed. Statistical significance was set at *p* < 0.05.

## 3. Results

**Figure 1:**
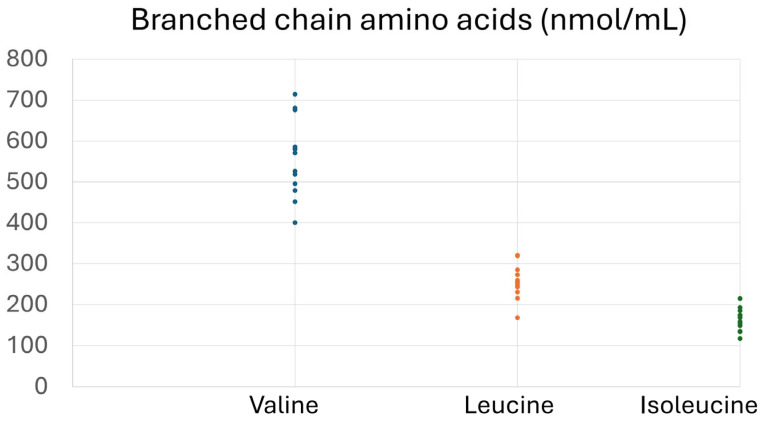

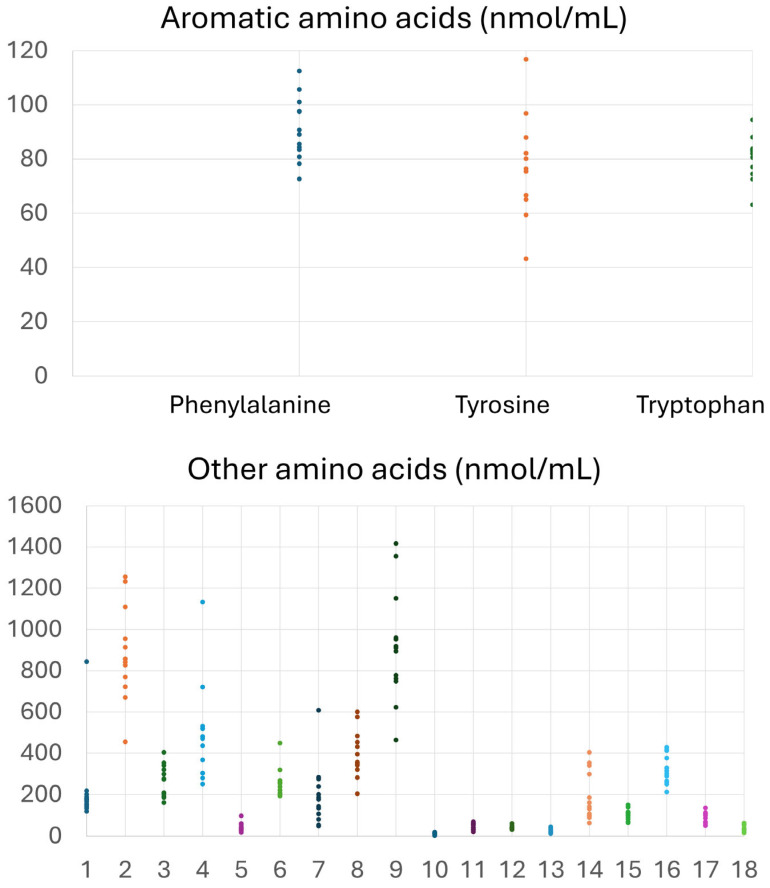
Concentrations (nmol/mL) of free amino acids in plasma samples of the participants at baseline. Other amino acids include the following: 1. Alo isoleucine, 2. Alanine, 3. Glutamic acid, 4. Glycine, 5. a-Aminobutyric acid, 6. Threonine, 7. Serine, 8. Proline, 9. Asparagine, 10. Thioproline, 11. Aspartic acid, 12. Methionine, 13. Hydroxyproline, 14. Glutamine, 15. Ornithine, 16. Lysine, 17. Hystidine, 18. Cystine.

Table 2 and Figure 1 present the descriptive characteristics of the subjects. As shown in Table 2, 61.5% of the subjects were males, the mean age was 56.3 ± 11.1 years, and all subjects had central obesity with BMI 35.6 ± 6.6 kg/m^2^. Mean glucose levels were above normal limits (105.9 ± 32.9 mg/dL), and HOMA-IR (4.36 ± 4.01) indicated significant insulin resistance. Mean TC (195.7 ± 33.2 mg/dL) and LDL (119.8 ± 31.1 mg/dL) were abnormal, and HDL was below the protective limit. Other biochemical indices that reflect hepatic, renal, and overall health exhibited normal levels. Free AAs (Figure 1) depict the baseline plasma levels of all amino acids, expressed in nmol/mL.

**Figure 2:**
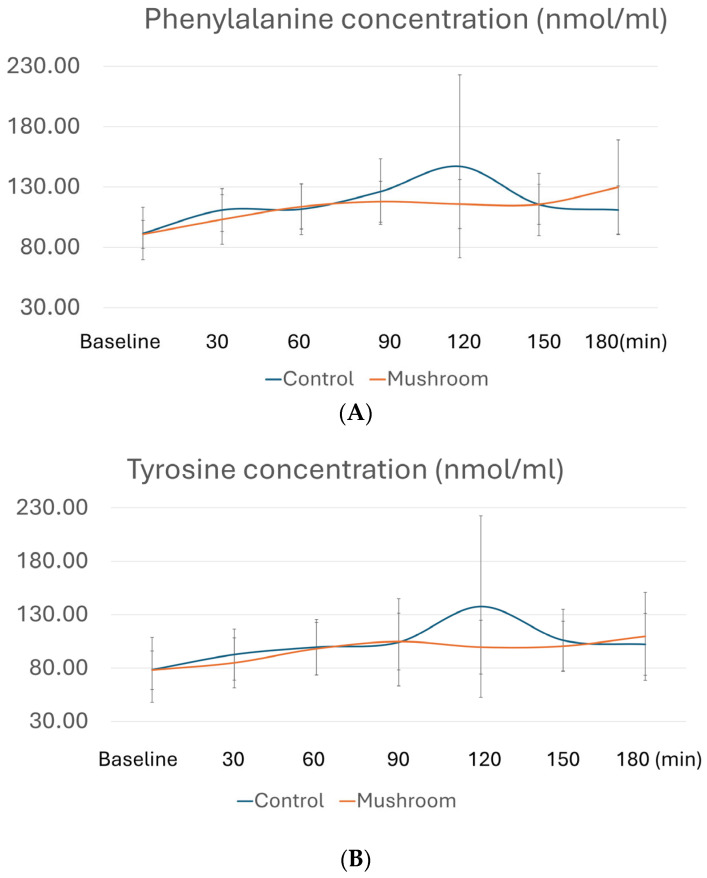

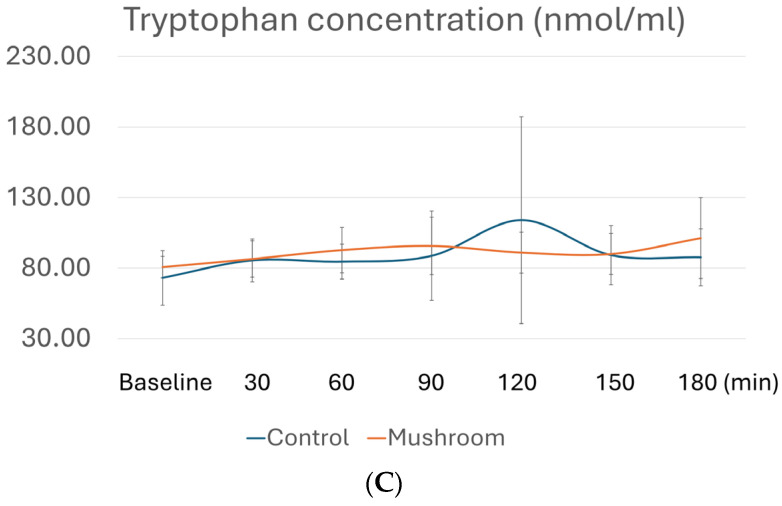
Postprandial amino acid profiles for (**A**) phenylalanine, (**B**) tyrosine, and (**C**) tryptophan at different timepoints. Concentrations of AAs in plasma after the control meal are depicted with blue, whereas after the meal with *P. eryngii* mushrooms, they are depicted in orange. For amino acid quantification, GC/MS analysis was conducted at baseline, 30 min, 60 min, 90 min, 120 min, 150 min, and 180 min after consumption of the two test meals, i.e., the mushroom meal and the control meal.

Table 3 presents the comparisons of postprandial AUCi of all AAs, whereas Figure 2 depicts postprandial levels of AAs with differences in AUCi. As shown in Table 3, consumption of the *P. eryngii* meal significantly affected AUCi for AAAs, namely phenylalanine, tyrosine, and tryptophan. More specifically, AUCi for all three AAAs was significantly higher after the consumption of the control meal compared to the *P. eryngii* meal (*p* = 0.027 for phenylalanine, *p* = 0.008 for tyrosine, and *p* = 0.003 for tryptophan), although no differences were observed between the two meals (*p* > 0.05) at any timepoint (Figure 2). No significant differences were observed between the two meals regarding the AUCi of all other amino acids (Table 3).

## 4. Discussion

Β-glucans in *Pleurotus* species exert several immunoregulatory activities due to their complex interaction with the immune system, which depends on the complexity of their molecular weight, branching degree, and presence of functional groups [19,20]. Also, several studies have shown the glucose and lipid regulatory effects of fungal β-glucans, mostly in animal models [21]. However, human studies show inconsistent results, most possibly due to their variability and the fact that β-glucans’ structure and branching with protein/peptide affect their conformation and may influence water solubility and biological activities [22]. Therefore, more research for the exploitation of the mechanisms under which β-glucans exert their metabolic regulatory properties is needed.

The complex profile of β-glucans in *P. eryngii* was most recently analyzed by Ellefsen and co-workers [4]. In detail, the alkali soluble Fucp-(1➔ , Xylp-(1➔ , ➔ 2)-Xylp-(1➔ , ➔ 3)-Galp-(1➔ , ➔ 6)-Galp-(1➔ , ➔ 2,6)-Galp-(1➔ , ➔ 3)-Manp-(1➔ , Glcp-(1➔ , ➔ 3)-Glcp-(1➔ , ➔ 6)-Glcp-(1➔ , and ➔ 3,6)-Glcp-(1➔ were identified. In our study, we showed that, owing to the b-glucans, postprandial AUCi of aromatic amino acids (AAAs: tyrosine, tryptophan, and phenylalanine) were significantly lower after consumption of a *P. eryngii* meal compared to a control meal. To the best of our knowledge, our study is the first that examines the in vivo potential of β-glucans in mushrooms to control the postprandial release of AAs into the bloodstream in people with central obesity and metabolic abnormalities. Overall, the results herein are similar to those in a cross-over study with a Mexican rural diet rich in dietary fibers, which induced a lower increase in phenylalanine, proline, and BCAAs compared to an urban diet in women from a Mexican rural population [23].

Recent research has shown that an altered plasma AAAs profile is observed in patients with metabolic abnormalities and cardiometabolic risk factors, such as BMI, blood pressure, lipids, and insulin [24,25,26]. Additionally, AAAs are higher in diabetic and obese patients compared to healthy individuals, with their levels being strongly associated with HOMA-IR and insulin [27]. In a Greek population of 100 middle-aged men, a BCAA/AAA-related pattern was associated with metabolic syndrome as well as with glucose and insulin levels, confirming the above associations in Greek individuals as well [28]. In our population, the postprandial decrease in AAA and the possible effect of the *P. eryngii* meal on their absorption coincide with the lower iAUC of glucose, ghrelin, hunger, and fullness shown previously in the same cross-over study [5], suggesting that postprandial alteration of AAAs is associated with glycemic and appetite regulation.

Protein source seems to affect postprandial AA profile, as a dairy meal induced higher postprandial isoleucine, tyrosine, and phenylalanine than fish, meat, or plant protein meals in a cross-over study with healthy individuals and four different isocaloric test meals [29]. Our findings support the proposed mechanism that protein digestibility is impaired by the presence of β-glucans due to their interaction with proteins. It is well known that AAs kinetics are affected by protein quality and quantity, and the presence of other dietary factors, such as fibers, that influence digesta passage kinetics via affecting viscosity [30,31]. In pigs that followed diets with increased concentrations of β-glucans, an effect on digesta physichochemical properties was observed, with increased mean retention time of digesta liquids and decreased digesta viscosity and protein digestibility in the stomach [30]. Studies investigating β-glucan release from fungal and plant cell walls showed that cooking before digestion increases β-glucan release from mycoproteins and barley bran, maybe through influencing their physicochemical properties, and protein release in white button mushrooms, although lower protein release was seen in digested oak flakes [31,32]. Interestingly, the gelatinized network of starch and β-glucans, presented in scanning electron microscopy in cooked barley, could explain the increased viscosity due to cooking and the blocking in protein release. Release of β-glucans from cell walls could be different in mushrooms and plants due to a different origin and cell wall structure [33].

The main strengths of this study include its cross-over design, where samples serve as their own controls, as well as the randomization in the order of meals that were consumed by the participants. Also, the incorporation of GC–MS analysis for the measurement of plasma free amino acids offers a very reliable quantitation.

Regarding the study limitations, we acknowledge the relatively small sample size and the sex imbalance that may have influenced our results. Although an effect on other amino acids, such as the BCAAs, might be expected based on their roles in obesity and abnormal metabolism, our results revealed significant findings exclusively in AAAs. This may be due to various factors such as the relatively low sample size or other biological implications, such as the different genetic backgrounds and intervariability of metabolic pathway regulations between subjects, which could have affected the detection of changes in the BCAAs.

## 5. Conclusions

Our results demonstrate the good performance of β-glucans present in *P. eryngii* mushrooms as potential modulators of AA release into the bloodstream. To the best of our knowledge, this is the first human study showing that β-glucans present in mushrooms affect protein digestion and the release of free AAs into the blood in central obesity. However, the small sample size could be considered as a limitation in generalizing the results, and in the future, similar studies in larger samples should be conducted. From a public health perspective, a dietary recommendation could be to add mushrooms to the meal of people with obesity and metabolic disorders to control the postprandial increase in free AAs in the blood and, further, to prevent insulin resistance. Beyond that, from a technological point of view, we consider the present results to be the starting point of research for the development of products aimed at influencing proteolysis through mushroom β-glucan enrichment.

## Figures and Tables

**Table 1:** Nutritional composition of the two experiments.

	Protein (g)	Fat (g)	Available Carbohydrates (g)	Energy Content (Kcal)
**Control meal**	23.0	12.7	55.4	427.9
***P. eryngii* meal**	25.5	13.3	52.6	432.1

**Table 2:** Anthropometric and biochemical characteristics of the study sample at baseline.

Characteristics	Baseline (N = 13)	
Gender	N	%
Males	8	61.5
Females	5	38.5
	Mean	SD
Age (years)	56.3	11.1
ΒΜΙ (kg/m^2^)	35.6	6.6
WC (cm)	115.8	14.0
HC (cm)	116.4	10.4
Fat (%)	38.9	8.9
Glucose (mg/dL)	105.9	32.9
Insulin (μU/mL)	14.8	8.6
HOMA-IR	4.36	4.0
ΤC (mg/dL)	195.7	33.2
TG (mg/dL)	129.4	61.6
HDL (mg/dL)	47.6	8.2
LDL (mg/dL)	119.8	31.1
AST (U/L)	21.5	7.1
ALT (U/L)	26.4	16.0
γ-GT (U/L)	25.2	15.5
ALP (U/L)	61.3	16.1
LDH (U/L)	178.3	18.9
Urea (mg/dL)	42.6	20.4
Uric acid (mg/dL)	6.1	1.6
Creatinine(mg/dL)	0.9	0.4
Iron (μg/dl)	82.7	11.9
Ferritin (ng/mL)	125.9	92.8
CRP (mg/L)	2.9	1.4

Continuous variables are presented as mean ± standard deviation (SD), and qualitative variables are presented as counts (%). BMI: body mass index, WC: waist circumference, HC: hip circumference, HOMA-IR: Homeostatic Model Assessment for Insulin Resistance, TC: total cholesterol, TG: triglycerides, HDL: high-density lipoprotein, LDL: low-density lipoprotein, AST: aspartate aminotransferase, ALT: alanine transaminase, γ-GT: γ-glutamyl transferase, ALP: alkaline phosphatase, LDH: lactate dehydrogenase, CRP: C-reactive protein.

**Table 3:** Postprandial AUCi of amino acids after the consumption of the two meals.

	Group		
	Control Meal	*P. eryngii* Meal	
	Mean ± SD	Mean ± SD	*p*-value
Alanine AUCi	23,101.1 ± 19,965	12,856.8 ± 26,935.7	0.209
Valine AUCi	14,862.1 ± 16,860.8	12,222.1 ± 12,138.5	0.417
Leucine AUCi	12,073.6 ± 10,153	9820.8 ± 5852	0.206
Aloisoleucine AUCi	1273.8 ± 29,265.5	−430.1 ± 31,845	0.898
Isoleucine AUCi	9945.1 ± 9300.5	−226.6 ± 31,473.2	0.307
Phenylalanine AUCi	5046.8 ± 2531.1	3889.6 ± 1773.2	**0.027**
Tyrosine AUCi	4959.9 ± 2222	3382.5 ± 1658.8	**0.008**
Tryptophan AUCi	3517.8 ± 1810.6	1887.6 ± 1163.8	**0.003**
Glutamic acid AUCi	12,632.4 ± 14,284.6	6774.9 ± 12,560.9	0.348
Glycine AUCi	3201 ± 10,956	−286 ± 12,271.6	0.369
a-Aminobutyric acid AUCi	−62.7 ± 1896.4	−334.4 ± 2389.2	0.502
Threonine AUCi	7032.3 ± 10,461	7390.9 ± 9517.2	0.937
Serine AUCi	8248.9 ± 10,030.9	7465.7 ± 11,921.8	0.884
Proline AUCi	35,099.9 ± 18,684.6	29,110.4 ± 13,137.3	0.313
Asparagine AUCi	45,248.5 ± 36,773	39,271.1 ± 31,880.2	0.667
Thioproline AUCi	247.5 ± 1201.2	345.4 ± 810.7	0.822
Aspartic acid AUCi	742.5 ± 1790.8	663.3 ± 1716	0.856
Methionine AUCi	1659.9 ± 1199	1446.5 ± 1266.1	0.556
Hydroxyproline AUCi	1115.4 ± 1558.7	1049.4 ± 1148.4	0.909
Glutamine AUCi	34,790.8 ± 46,304.5	31,037.6 ± 48,439.6	0.652
Ornithine AUCi	3204.3 ± 2879.8	2979.9 ± 1919.5	0.728
Lysine AUCi	10,063.9 ± 6319.5	7527.3 ± 4842.2	0.160
Histidine AUCi	964.7 ± 3801.6	927.3 ± 3124	0.967
Cystine AUCi	139.7 ± 1404.7	500.5 ± 1018.6	0.492

Data are presented as mean ± standard deviation (SD). *p*-value stands for the comparison between the two meals via paired *t*-test. Statistical significance was set at *p* < 0.05 and significant *p* are in bold. AUCi: area under the curve with respect to increase.

## Data Availability

The original contributions presented in the study are included in the article/Supplementary Materials, further inquiries can be directed to the corresponding author.

## Supplementary Materials

The following supporting information can be downloaded at: https://www.mdpi.com/article/10.3390/foods14152649/s1, Table S1: Retention times (Rt) and *m*/*z* of ions used for the selective ion monitoring of amino acids.

## Abbreviations

The following abbreviations are used in this manuscript:

ALT	Alanine transaminase
AAs	Amino acids
ALP	Alkaline phosphatase
AUCi	Area under the curve with respect to increase
AAAs	Aromatic amino acids
AST	Aspartate aminotransferase
BMI	Body mass index
BCAAs	Branched-chain amino acids
CRP	C-reactive protein
γ-GT	γ-glutamyl transferase
GC/MS	Gas chromatography–mass spectrometry
HDL	High-density lipoprotein
HC	Hip circumference
HOMA-IR	Homeostatic Model Assessment for Insulin Resistance
LDH	Lactate dehydrogenase
LDL	Low-density lipoprotein
*P. eryngii*	*Pleurotus eryngii*
TC	Total cholesterol
TG	Triglycerides
T2D	Type 2 diabetes
WC	Waist circumference
