# Supplementary material for: The Performance of Pleurotus eryngii β-Glucans on Protein Digestion and the Release of Free Amino Acids in the Bloodstream of Obese Adults
Source: Foods. 2025 Jul 28;14(15):2649. doi: 10.3390/foods14152649 (PMC12346340; doi:10.3390/foods14152649)
Supplement: Supplementary file 1 [file foods-14-02649-s001.zip › foods-3775483-supplementary.pdf]

Supplementary material related to:

The Performance of *Pleurotus eryngii* β-Glucans on Protein Digestion and the Release of Free Amino Acids in the Bloodstream of Obese Adults

Charalampia Amerikanou [1,*], Stamatia-Angeliki Kleftaki [1], Aristea Gioxari [2], Dimitra Tagkouli [1], Alexandra Kasoura [1], Stamatia Simati [3], Chara Tzavara [1], Alexander Kokkinos [3], Nick Kalogeropoulos [1] and Andriana C. Kaliora [1]

[1] Department of Nutrition and Dietetics, School of Health Science and Education, Harokopio University, 17676 Athens, Greece; matina.kleftaki@gmail.com (S.-A.K.); dtagkoul@hua.gr (D.T.); alexkasoura@gmail.com (A.K.); htzavara@med.uoa.gr (C.T.); nickal@hua.gr (N.K.); akaliora@hua.gr (A.C.K.)

[2] Department of Nutritional Science and Dietetics, School of Health Science, University of the Peloponnese, Antikalamos, 24100 Kalamata-Messinia, Greece; a.gioxari@uop.gr

[3] First Department of Propaedeutic Internal Medicine, Laiko General Hospital, National and Kapodistrian University of Athens School of Medicine, 11527 Athens, Greece; simatistemi@gmail.com (S.S.); akokkinos@med.uoa.gr (A.K.)

* Correspondence: camer@hua.gr or amerikanou@windowslive.com; Tel.: +30-2109549226; +30-6947526539

Table S1. Retention times (Rt) and m/z of ions used for the selective ion monitoring of amino acids.

Target Compounds	Abbreviation	Rt (min)	Ions (m/z)*
Alanine	Ala	1.063	130, 88
Glycine	Gly	1.163	116, 207
Valine	Val	1.357	158, 116
Norvaline (internal standard)	Nva	1.478	158,72
Leucine	Leu	1.623	172, 86
Isoleucine	Ile	1.622	172, 130
Threonine	Thr	1.841	160, 101
γ-Aminobutyric acid	GABA	1.850	130, 86, 112, 172
Serine	Ser	1.866	146, 203
Proline	Pro	1.935	156, 243
Asparagine	Asn	2.038	155, 69
Thioproline	Thp	2.590	174, 147
Aspartic acid	Asp	2.597	216, 130
Methionine	Met	2.619	203, 277
4-Hydroxyproline	4Hyp	2.969	172, 86, 130
Glutamic acid	Glu	2.971	230, 170
Phenylalanine	Phe	2.982	206, 190
Glutamine	Gln	3.614	84, 187
Ornithine	Orn	4.019	156, 70
Lysine	Lys	4.292	170, 128
Histidine	His	4.468	282, 168
Tyrosine	Tyr	4.759	206, 107
Tryptophan	Trp	5.028	130
Cystine	Cys	5.763	248, 216

*: User's Manual; Phenomenex® EZ:faast™ Free (Physiological) Amino Acid Analysis by GC-MS for Agilent GC/MS instruments.
